# Modulation of the Oxygenation State and Intracellular pH of Erythrocytes by Inositol‐Trispyrophosphate Investigated by ^31^P NMR Study of 2,3‐DPG

**DOI:** 10.1111/jcmm.70343

**Published:** 2025-01-19

**Authors:** Sabina Koj, Tomasz Niedziela, Joanna Rossowska, Jean‐Louis Schmitt, Jean‐Marie Lehn, Claude Nicolau, Claudine Kieda

**Affiliations:** ^1^ Hirszfeld Institute of Immunology and Experimental Therapy Polish Academy of Sciences Wroclaw Poland; ^2^ ISIS—University of Strasbourg Strasbourg France; ^3^ NormOxys Inc. Boston Massachusetts USA; ^4^ Friedman School of Nutrition Science and Policy Tufts University Boston Massachusetts USA; ^5^ Centre for Molecular Biophysics, UPR CNRS 4301 Orleans France; ^6^ Laboratory of Molecular Oncology and Innovative Therapies Military Institute of Medicine—National Research Institute Warsaw Poland

**Keywords:** 2,3‐diphosphoglycerate, 31P NMR, erythrocyte, hypoxia, *myo*‐inositol trispyrophosphate, oxygen, pH

## Abstract

The hypoxic microenvironment is crucial for tumour cell growth and invasiveness. Tumour tissue results from adaptation to reduced oxygen availability. Hypoxia first activates pro‐angiogenic signals for alleviation. Pathologic, tumour angiogenesis maintains hypoxia, impairing treatment outcomes. Vessel normalisation requires physioxia. Oxygen delivery by red blood cell (RBC) carrying haemoglobin (Hb) is enhanced by *myo*‐inositol trispyrophosphate (ITPP), an effector of oxygen transport by RBCs. Altering glycolytic activity, it lowers intracellular pH and increases oxygen release from Hb. ^31^P NMR tracking of 2,3‐diphosphoglycerate (2,3‐DPG), allosteric effector of Hb and non‐penetrating anion in RBCs, reports on erythrocytes internal environment. ^31^P resonances of 2,3‐DPG are pH‐sensitive, their positions indicate the oxygenation state of RBCs and interactions with effectors such as ITPP. Here we show in vitro and in vivo, that modifying Hb activity through band‐3 anion transporter, ITPP enhances oxygen release and controls RBC internal pH. Its blood availability validates applicability of ITPP‐based strategies.

**Abbreviations**
2,3‐DPG: 2,3‐diphosphoglycerate
DIDS: 4,4′‐diisothiocyanatostilbene‐2,2′‐disulfonic acid
Hb: haemoglobin
ITPP: *myo*‐inositol trispyrophosphate
RBC: red blood cell

## 1 Introduction

Hypoxia is a hallmark of growing tumours. Hypoxic stress affects tumour cells and the various cells that constitute the tumour tissue. In such an environment, cancer cells proliferate and invade distant sites because of their adaptability to reduced oxygen supply. The lowering of pH, along with the overall cellular and molecular reactions at the tumour site, contributes to tumour aggression, which is a consequence of hypoxia.

Among the deleterious effects of hypoxia, signalling to endothelial cells initially activates angiogenesis to provide blood flow for oxygen and nutrient supply to the tumour site. However, tumour angiogenesis is pathological, responding to transcription of hypoxia‐inducible factor‐1 (HIF‐1) into pro‐angiogenic factors such as VEGF‐A, which are constantly overproduced by hypoxic tumour cells [1]. Pathological angiogenesis cannot restore a physiological oxygen partial pressure (pO_2_), and such chronic hypoxia impairs treatments efficacy, raising the need for strategies to restore a physiological level of pO_2_ [2].

Vasculature remodelling into normal vessels appears highly promising to ensure efficient blood flow necessary to deliver anticancer drugs via chemo‐ and immunotherapy and aid radiotherapy. Normalisation of the vasculature is possible through increased oxygen delivery by haemoglobin (Hb), which reduces VEGF‐A overproduction, as shown by hypoxia alleviation [3] and cell‐gene therapy [4]. This leads to lower vessel permeability and oedema, vessel pruning, and modifications in the biological, immunological, and biochemical composition of the tumour microenvironment, which are the main achievements of successful cancer treatment [5].

The discovery by Benesch et al. [6] of allosteric effectors and their functions in birds allowed the modulation of Hb affinity for oxygen. In 1979, Gersonde and Nicolau reported the encapsulation of inositol hexaphosphate (IP_6_) in intact red blood cells (RBCs) [7] and the modulation of Hb affinity for O_2_. Another allosteric effector of Hb to fulfil this condition is myo‐inositol trispyrophosphate (ITPP). ITPP enhances oxygen release from RBC Hb in vitro, [8, 9, 10] thus, restoring physioxia in hypoxic tissues as demonstrated by biomarkers such as HIF‐1, VEGF and caspase 3 in vivo [11, 12, 13, 14].

The action of ITPP in vivo on tumour angiogenesis leads to blood vessel normalisation [3, 13, 15]. ITPP's molecular mechanisms for normalising vessels rely on facilitating oxygen release and activating the tumour suppressor PTEN, [3] a major controller of angiogenesis [16]. PTEN activation by ITPP is direct and favoured by intracellular conditions [17].

This two‐step mechanism of vessel normalisation is initiated by oxygen release and is stabilised upon PTEN activation. Potential consequences for new treatments of hypoxia‐dependent diseases such as cancer [14], diabetes, heart‐related pathologies [18, 19, 20, 21], and respiratory distress, as seen in virus induced hypoxia as Coronavirus Disease 2019 (COVID‐19) [22] require an understanding of the molecular mechanisms by which ITPP enters RBCs and modifies the biochemical pathways of efficient O_2_ release [9].

Glycolytic enzymes in RBCs are activated upon oxygen deprivation by displacement from their binding site on band‐3, thus, hypoxia ultimately lowers the pH in RBCs. Consequently, the oxygenation state of Hb and its interaction with its natural allosteric effector diphosphoglycerate (2,3‐DPG) is a process in which pH is a key parameter.

Indeed glyceraldehyde 3‐phosphate dehydrogenase (GAPDH) binds to the cytosolic N‐terminus of band‐3 competitively to binding with the deoxygenated form of Hb, in response to cellular needs and antioxidative requirements, the metabolism of glucose is directed between glycolysis and the pentose phosphate pathway. Thus band 3 interactions lead the RBC molecular switch between oxygenated/deoxygenated state [23].

The band‐3 is an ion transporter that exchanges anions across the red cell membrane that is also affected by competitive stilbenedisulfonate inhibitors, such as DIDS (4,4′‐diisothiocyanatostilbene‐2,2′‐disulfonic acid) [24, 25]. As the band‐3 is a major integral protein of the red cell and fill a critical role by transporting oxygen, as well as it mediates chloride‐bicarbonate exchange and provides a binding site for glycolytic enzymes, Hb, and the skeletal proteins, the inhibition of this important protein may have strong impact on the RBC function [26]. Consequently, the action of the RBC‐affecting factors can lead to disruption of physiological processes, involving catalysis of the transmembrane Cl^−^/HCO_3_^−^ exchange, CO_2_ excretion, oxygen release, membrane skeleton binding, regulation of Hb activity, homeostasis and redox balance in RBC.

2,3‐DPG is an abundant component of erythrocytes, with its intracellular concentration on par with that of Hb. The high natural abundance of the ^31^P makes it suitable for NMR tracking of phosphorous‐containing biological compounds such as 2,3‐DPG. Encapsulated 2,3‐DPG in intact RBCs constitutes non‐penetrating anions. Therefore, 2,3‐DPG can be used as a marker of the inner environment of erythrocytes, reflecting their internal pH and oxygenation state.

^31^P resonances of 2,3‐DPG by NMR allow the assessment of the state of Hb and its ability to interact with oxygen [27, 28, 29, 30, 31, 32, 33]. The resonances of the phosphates are differently pH‐sensitive and may be used to report pH changes inside RBCs in relation to Hb oxygenation state. Previously, this method was used to estimate blood hypoxia as a reporter of hypoxia‐dependent diseases [34].

ITPP enhances Hb capacity to release oxygen, appearing capable of restoring physioxia in hypoxia‐injured tissues 10 and counterbalancing the effects of pH lowering, which explains the restricted pH range in which ITPP effects are detected. Early ^31^P NMR studies showed that the strong allosteric effector IP_6_ could be introduced into RBCs without affecting their properties and influenced oxyHb dissociation [32]. The ^31^P NMR spectrum of incorporated IP_6_ remained identical to that of the adduct of IP_6_ with stripped oxyHb.

This paper describes the use of ^31^P NMR analysis of the ITPP interaction with intact RBCs and, based on the described effect on band‐3 [9] ion transporter in the RBC membrane, its control of the glycolysis pathway, which results in pH lowering. ITPP mechanism of action is assessed primarily by its effect on RBC internal pH. However, pH changes are not the sole cause of the chemical shift variation of 2,3‐DPG. They depend on the magnetic susceptibility in the microenvironment of RBCs as a direct result of an increasing amount of deoxyHb. Upon Hb deoxygenation, the deoxyHb binds strongly to 2,3‐DPG, causing a downfield shift of 3‐P & 2‐P resonances, which are larger than for the inorganic phosphates (P_i_). Moreover, the resonances broaden as the fraction of deoxyHb increases. Inactivation of band‐3 also nullifies the effects of ITPP on internal pH. The dynamic balance of the internal versus external pH and concentrations in vitro was validated in vivo by comparative NMR measurements for the presence of ITPP inside and outside the RBC in the blood.

## 2 Results

### 2.1 Estimation of the RBC Internal pH— ^31^P Resonances in 1D NMR Spectra of 2,3‐DPG and Their pH‐Dependence

**FIGURE 1:**
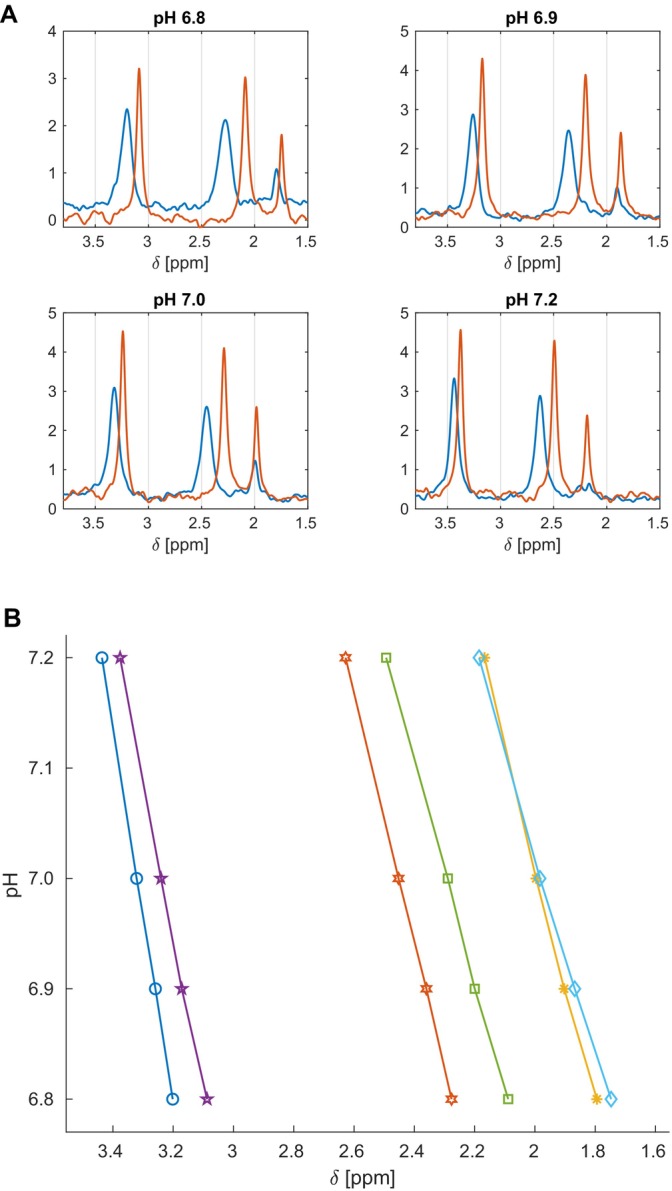
Chemical shift values of 3‐P(DPG), 2‐P(DPG) and Pi resonances in the 1D NMR ^31^P spectra of intact and lysed RBCs (A) and the pH dependence of their positions in the spectra (B) recorded at different pH values. Blood samples were suspended and washed using the 30 mM HEPES buffer with nominal pH values of 6.8, 6.9, 7.0 and 7.2. Lysis of the intact RBCs was performed by sonication. In the spectra, the blue lines indicate the 2,3‐DPG signals for the apparent internal pH, and the red lines correspond to the signals acquired after lysis in the same buffer, indicating the actual pH. The 256‐spectra were accumulated & averaged for each pH value. The peak‐picking was done using the Topspin ‘auto‐pick’ algorithm. The Stdev values were ≤ +/−0.01 (≤ +/−0.02 for Pi) and are not shown.

Intact RBCs were suspended in 30 mM HEPES‐buffered solutions with defined pH values: 6.8, 6.9, 7.0 and 7.2. NMR spectra of RBC suspensions were acquired (Figure 1A). Subsequently, RBCs were lysed by sonication, and NMR spectra of the lysed RBC solutions were recorded (Figure 1A). The pH values for the intact RBC suspensions and the lysed RBC were measured. The positions of 2‐P(DPG), 3‐P(DPG), and P_i_ resonance in the 1D NMR ^31^P spectra of both intact and lysed RBCs were compared and correlated with the pH values as measured in the respective buffer solutions.

A substantial change in the chemical shift values of 2‐P(DPG) and 3‐P(DPG) resonances in the 1D ^31^P NMR spectra was observed between the intact RBC suspensions and their lysed solutions in the same buffer. This indicates that the internal pH of the RBC differs from the pH of the buffer, reflecting the apparent pH of the internal microenvironment in the RBC. Moreover, the chemical shifts (δ) of 2‐P(DPG) and 3‐P(DPG) recorded in buffers with distinct pH values allow for the estimation of the RBC internal pH. The relationship between chemical shift differences and pH values (Figure 1B) before and after lysis of the RBCs exhibits near‐linear pH dependence. The chemical shift differences (Δδ) indicate a constant right shift after lysis, thus evidencing a lower internal pH in the RBCs.

Briefly, ^31^P resonances of the 3‐P and 2‐P of the 2,3‐DPG in the NMR spectra of the intact RBC at pH 6.8 were observed at ~3.20 and ~2.28 ppm respectively. After RBC lysis, these chemical shift values decreased to ~3.09 and ~2.09 ppm respectively (Figure 1A,B and Table S1). The chemical shift differences Δδ ~0.1 and Δδ ~0.2 ppm (up‐field shift) can be correlated with the pH lowering after lysis. Similar chemical shift changes of the 3‐P and 2‐P resonances of the 2,3‐DPG between the intact RBC and lysed RBC were demonstrated for all tested pH values in the 6.8–7.2 range.

Alterations of the phosphate chemical shift values reflect physiological changes of RBCs that accompany changes in the oxygenation state of Hb [28, 29]. The right shift (up‐field) correlates with [H^+^] increase after lysis, indicating more acidic conditions. Along with the shift value differences, signals intensities increased after lysis, demonstrating that RBCs are oxygenated. Both line narrowing (sharp signals) and lower chemical shift of the 2,3‐DPG ^31^P resonances after lysis confirm a higher H^+^ concentration alongside the oxygenated state of RBCs. This agreed with the observations by Labotka [28].

### 2.2 The 2,3‐DPG Resonances Measured by ^31^P NMR Indicate the RBC Internal Versus External pH Equilibria In Vitro. DIDS‐Mediated Inhibition of Band‐3 Influence on ITPP‐Induced pH Modification in RBC

The positions of ^31^P resonances in 1D NMR spectra are pH‐dependent. However, they also vary with the oxygenation state of RBCs, and these two processes cannot be resolved unequivocally. Standardised conditions allow to assumption that the RBC oxygenation state for any given sample was maintained during the measurements.

The reservoir of phosphates inside RBCs, 2,3‐DPG, provides distinct signals of ^31^P resonances. These are readily identifiable, along with those of free phosphates, and both intensity and position in the spectra correlate with RBC metabolic state. The positions of ^31^P signals from 2‐P(DPG), 3‐P(DPG) and, to a lesser extent, from free phosphate (P_i_), depend on the trans‐membrane ion‐exchange processes regulated by band‐3.

We devised 1D ^31^P NMR experiments to visualise the resonances of 2,3‐DPG in intact RBCs and investigate the effect of ITPP on the oxygenation state of RBCs in vitro. ITPP is known to shift the oxygenation balance in erythrocytes, facilitating oxygen release [3, 5, 8, 21, 35, 36, 37, 38]. As the oxygenation state and pH are also co‐dependent, we attempted to observe this using the changes in the ^31^P NMR profiles of the 2,3‐DPG in native RBCs compared with ITPP‐treated RBCs. Additionally, we analysed the role of band‐3 protein in the active transport of ITPP to RBCs and how DIDS influences its effects.

**FIGURE 2:**
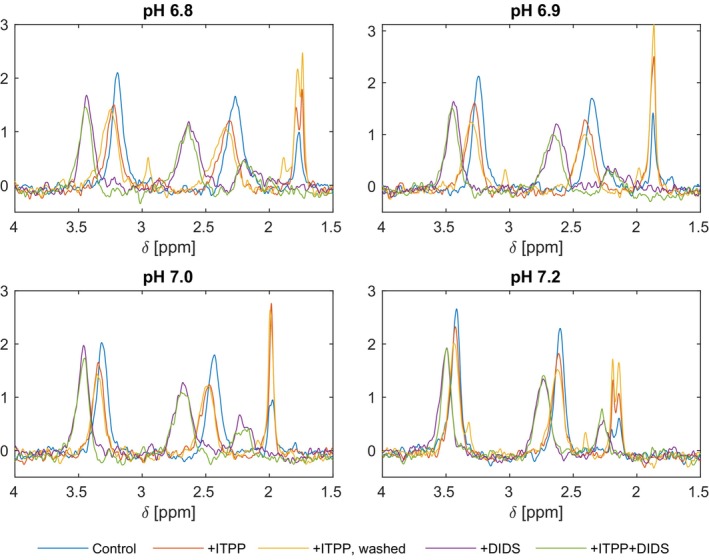
Chemical shift values of the 3‐P(DPG), 2‐P(DPG) and P_i_ resonances in the 1D NMR ^31^P spectra of intact RBC suspensions in buffers with pH value*s* of 6.8 (A), 6.9 (B), 7.0 (C) and 7.2 (D). Intact RBC samples were suspended in 30 mM HEPES buffer at pH value*s* of 6.8, 6.9, 7.0 and 7.2. The spectra were acquired for ITPP‐treated RBCs and for ITPP‐treated RBCs in the presence of the band‐3 inhibitor (DIDS). Data are mean values from *n* = 6 samples. Spectra of the intact RBC suspensions and in the presence of DIDS alone were recorded at the corresponding pH‐values as controls.

Briefly, Figure 2 shows that at pH 6.8, ^31^P resonances of the 3‐P and 2‐P of 2,3‐DPG in the NMR spectra of intact RBCs were observed at ~3.19 and ~2.27 ppm respectively. After RBC treatment by ITPP, signal positions moved to higher chemical shift values, ~3.22 and ~2.32 ppm respectively (Table S2). The chemical shift differences of Δδ ~0.05 ppm (downfield shift) indicate a rise of pH. Moreover, RBC spectra signals broadened with ITPP treatment, while the presence of DIDS resulted in signals sharpening back; they also strongly shifted to higher chemical shifts, ~3.44 ppm. Such differences were also observed, but to a lesser extent at higher pH values (pH 7.0 and pH 7.2), where signal broadening was still observed.

In vitro RBC treatment by ITPP showed that 3‐P and 2‐P signals of 2,3‐DPG moved to higher chemical shifts values (Δδ ~0.05 ppm). This is opposite to Figure 1 data, where up‐field shift and sharper signals indicated that pH lowered after lysis, demonstrating acidification and oxygenated RBCs [28]. In contrast, the down‐field shift means that pH became less acidic when the RBCs were treated with ITPP. Moreover, the down‐field shift indicated that 2,3‐DPG was bound to deoxyHb. The ^31^P signals broadened distinctly, indicating that deoxyHb levels increased in RBCs.

In summary, down‐field changes of 2,3‐DPG signals and the NMR line broadening indicated a pH increase, elevated deoxyHb in ITPP‐treated erythrocytes, and the presence of 2,3‐DPG bound to deoxyHb. These conditions accompany deoxygenation and oxygen release from RBCs.

Additionally, simultaneous RBC treatment with ITPP and DIDS showed that DIDS abolished all ITPP effects on RBCs. DIDS strongly influenced the 3‐P and 2‐P signals, moving them to higher chemical shift values, from ~3.19 and ~2.27 ppm – ~3.44 and ~2.63 ppm respectively. The effects on pH, along with a lack of line broadening, indicated that ITPP did not elevate the amount of deoxyHb in RBCs when the inhibitor of the membrane‐associated channel protein band‐3 was present, thus, ITPP was not transported across the RBC membrane.

### 2.3 Effects of ITPP on the RBC—In Vivo Experiments

**FIGURE 3:**
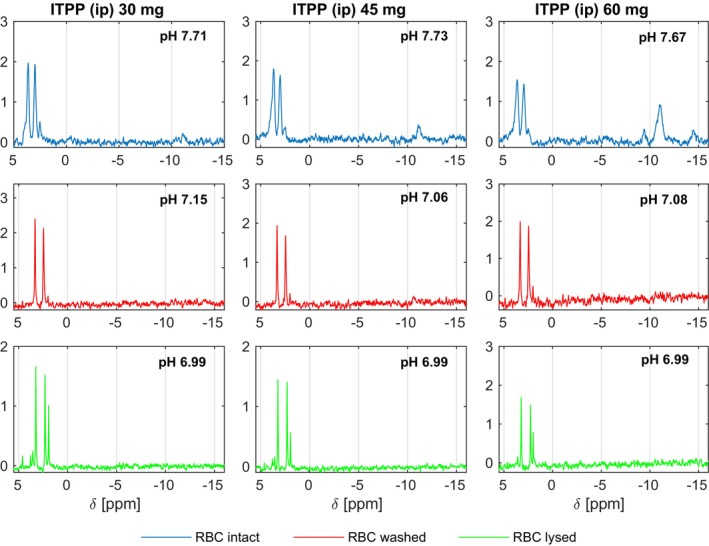
In vivo analysis of the pH‐dependence of 3‐P(DPG), 2‐P(DPG) and P_i_ chemical shifts in the 1D NMR ^31^P spectra of RBCs. Mice were inoculated intraperitoneally with increasing doses of ITPP: 30, 45 and 60 mg. After 1 h, blood samples were collected from each individual mouse (*n* = 6), and the pH was measured. The spectra were recorded for the intact RBCs (blue lines) and re‐recorded after the washing step (red lines), and after lysis (green lines). 30 mM HEPES, pH 7.0 was used as a washing buffer.

The in vivo effects of ITPP were analysed using a mouse model. Animals were inoculated i.p. with increasing doses of ITPP (30, 45 and 60 mg). Blood samples were collected and the initial pH was measured. The ^31^P 1D NMR spectra were recorded for the intact RBC suspension, re‐recorded after washing, and after lysis in washing buffer. Changes in the chemical shift values of 3‐P(DPG), 2‐P(DPG), and P_i_ spectra were observed and correlated with the ITPP doses used for inoculation. Intraperitoneally injected ITPP was detected in the blood after 1 h (Figure 3).

The observed effects were dose‐dependent. The resonances of 2,3‐DPG in the RBC were down‐field shifted in the presence of ITPP. The effect diminished after washing (HEPES buffer, pH 7.0), and the internal to external pH change was visible after RBC lysis. This was visible after lysis since no pO_2_ elevation occurs inside RBCs, which maintains a balance by controlling the activity of glycolytic enzymes.

ITPP was detected in the blood of intraperitoneally inoculated animals. This observation is very important as it confirms further availability of ITPP for endothelial PTEN and its activation [3, 17]. The comparison of the integral values for this region recorded for ITPP‐calibrated standard solution (a set of signals in the chemical shift range δ = −9 to −16 ppm) with these for the same region in the ^31^P 1D NMR spectra of blood samples allowed for a rough estimate of the ITPP blood content as ~1% of the intraperitoneally injected ITPP (60 mg). However, for the lower doses (30, 45 mg) the signals were below the ^31^P 1D NMR detection limits.

**FIGURE 4:**
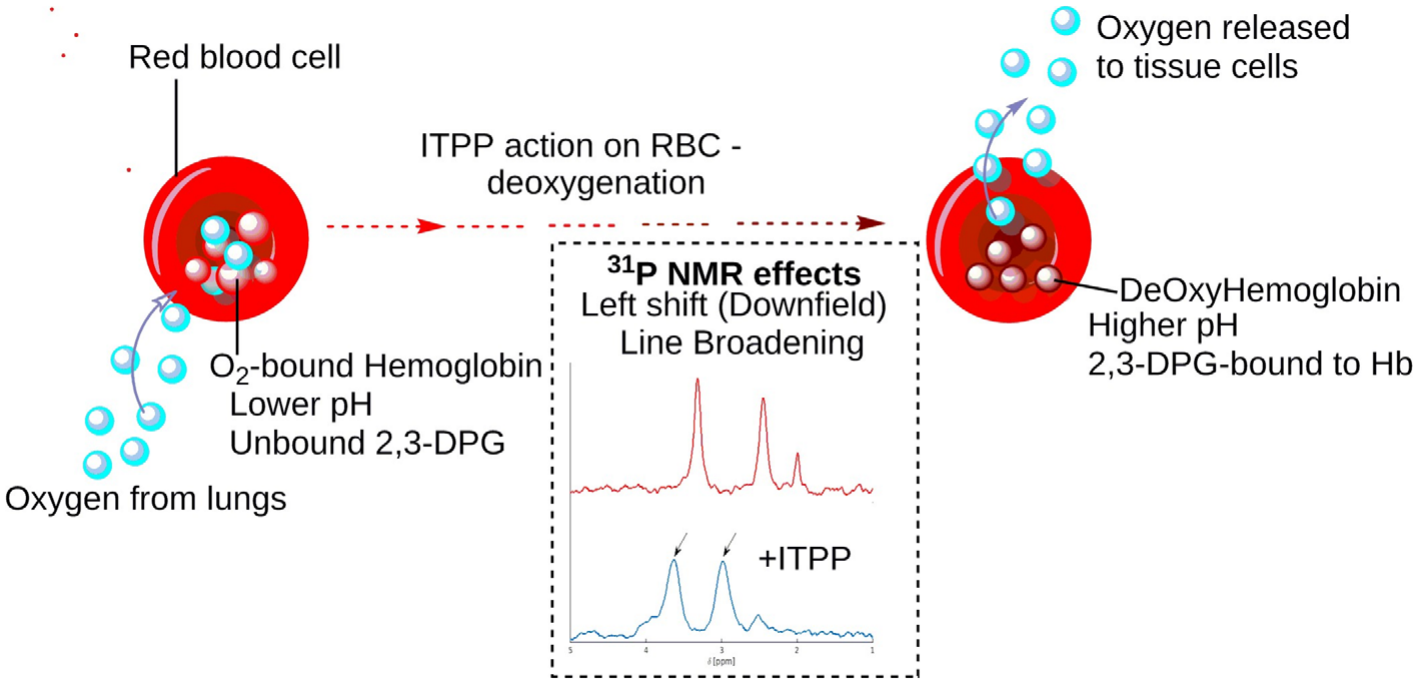
Relative changes that occur during the deoxygenation and oxygenation of RBCs. Potential mechanism of ITPP action on RBC. Listed are differences of conditions in oxygenated RBC (Oxy‐RBC) and deoxygenated RBC (Deoxy‐RBC) measured by ^31^P NMR spectroscopy. Differences in NMR signals of 2,3 DPG phosphates indicate pH changes and the binding state of Hb.

^31^P resonances of the 3‐P and 2‐P of the 2,3‐DPG in the NMR spectra of the RBCs at pH 7.0 are observed at ~3.32 and ~2.45 ppm respectively (Figure 1). After treatment of mice with ITPP (30 mg), the ^31^P spectra of isolated RBCs contained signals at ~3.63 and ~2.99 ppm respectively (Figure 3, Table S3). The down‐field shifts of the phosphate resonances are relatively strong (Δδ ~0.3 ppm for 3‐P and ~0.5 ppm for 2‐P), indicating that the pH became less acidic and that 2,3‐DPG was bound. Moreover, a strong line broadening was observed in the spectra. The effects were similar to those observed in vitro, confirming that in the presence of ITPP, the amount of deoxyHb increases in RBCs. The pH rise accompanies the deoxygenation of the cells. Summarising, the differences in NMR signals of 2,3‐DPG phosphates between the intact RBCs and the ITPP‐treated RBCs indicated pH changes and binding state of Hb, suggesting the potential mechanism of action. ITPP, as an allosteric effector of Hb, favours the deoxygenated state of erythrocytes (Figure 4). Thus, the ITPP‐induced oxygen release can be effectively delivered from the bloodstream to hypoxic tissues.

After washing and lysis of the RBC isolated from ITPP‐treated mice, the 3‐P and 2‐P signals in the ^31^P spectra shifted to ~3.56 and ~2.31 ppm, respectively (Figure 3 and Table S3). As ITPP interacts at the RBC membrane and enters the cells, the washing/lysis procedure might have reduced its effect on the RBCs.

Signal at ~2.0 ppm in the NMR spectra of RBC at pH 7.0 (Figures 1 and 2) corresponds to native inorganic phosphates (P_i_) present in RBC cytoplasm. The signal of P_i_ shifts up‐field and becomes more intense upon lysis compared with intact RBCs (Figures 1 and 2). This signal differs from the 2,3‐DPG ones. Opposite to organic phosphate, P_i_ does not bind Hb significantly intracellularly; therefore, this interaction cannot account for the observed differences in the chemical shift [28]. The increased intensity of the NMR signals of P_i_ in the ITPP‐treated RBCs may reflect an increase in intracellular pH with deoxygenation, consistent with deoxyHb being a stronger base than oxyHb [39, 40]. The pH increased from 7.15 to 7.29 upon deoxygenation [28].

Intracellular phosphates resonances are characteristically broadened [28], therefore the changes of line‐broadenings in the [31] P spectra of RBCs (Figure 2) indicated that 2,3‐DPG, present inside RBCs, bound to deoxyHb while inorganic phosphates (P_i_) are extracellular. Sharp signals at ~1.94 ppm in the ^31^P spectra of isolated RBCs (Figure 3) also correspond to the unbound extracellular phosphate (P_i_).

### 2.4 ITPP Interactions With RBCs and Limits of Detection by NMR Techniques

A comparison of the NMR spectra of ITPP‐treated RBCs and corresponding spectra recorded after washing indicated some residual ITPP. However, the consistency of the ^31^P chemical shift values of the ITPP throughout the procedure—no discrepancy between internal and external ITPP signals—did not allow for the unequivocal localisation of the remaining ITPP. As the spectra were acquired using an identical setup, estimation of the amount of ITPP remaining in these samples was done by comparison of the integrals. Washed RBC samples retained approximately 12%–15% of the initial ITPP amount used for stimulation in vitro (3 mg/mL). In in vivo experiments, the ITPP signals were not detected in the RBC preparations.

**FIGURE 5:**
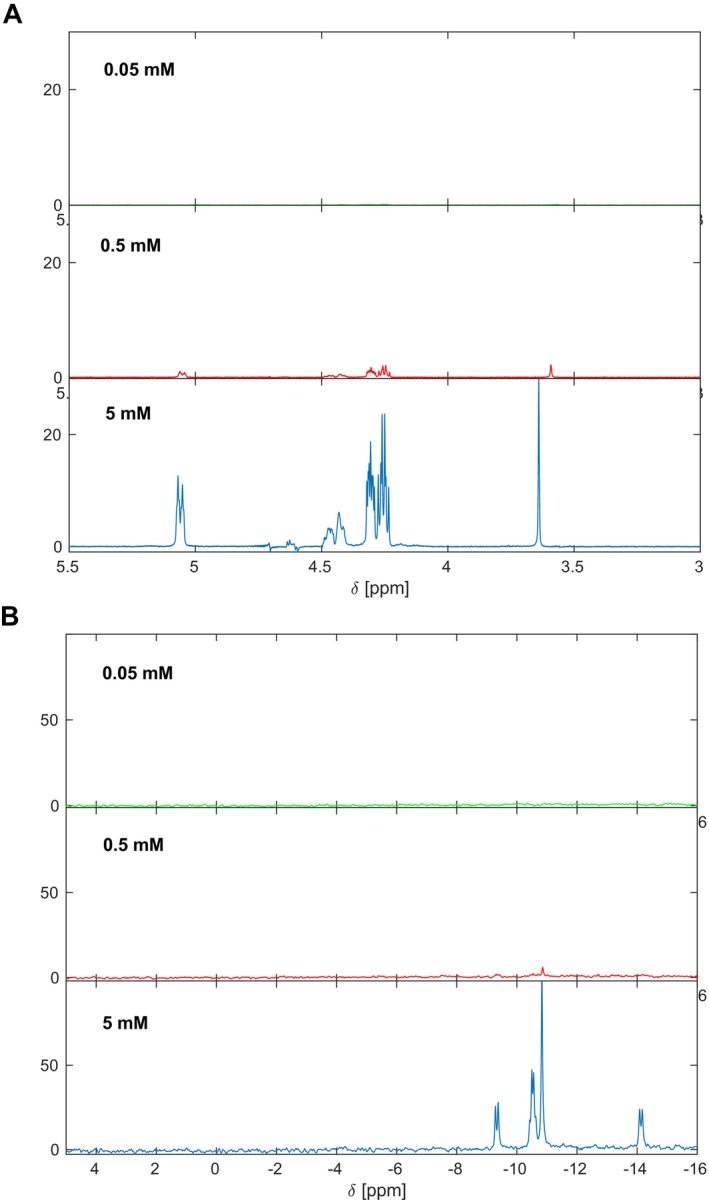
NMR detection limit for ITPP in solution. ^1^H spectra (A) and ^31^P spectra (B) were acquired in D_2_O* at 298 K for three 10‐fold dilutions of ITPP (5, 0.5 mM, and 50 μM). *The HEPES‐containing buffer was not used to avoid the large resonances in ^1^H NMR that compromise the dynamic range of the measurements.

To find the practical NMR detection limit of ITPP, it was serially diluted (1.5 mg, 150 and 15 μg in 0.5 mL) in D_2_O. Both ^1^H and ^31^P spectra were collected (Figures 5A and 5B respectively). The ^1^H spectra indicated that effectively, ITPP at ~0.5 mM concentration is the detection limit by NMR. ^31^P spectra were weaker, as the ^31^P sensitivity is approximately 7% of ^1^H. All data indicated that RBC‐internalised ITPP cannot be directly detected using ^31^P NMR. This can be explained by the transmembrane ion exchange dynamics operated by band‐3 under the experimental conditions and limited sensitivity of ^31^P NMR detection.

## 3 Discussion

Modulation of erythrocyte functions in response to physiological conditions maintains oxygen homeostasis in tissues. Oxygen supply through Hb‐mediated oxygen uptake and release is strictly controlled to prevent hypoxia and other oxygenation‐dependent pathologies resulting from microcirculation impairment. The respiratory gas cycle provides adequate oxygen and nutrient delivery for local metabolic demand. The proper blood flow and blood oxygen content ensure tissue oxygenation. The control of oxygen delivery, microvascular origins of hypoxia and Hb‐mediated regulation of microcirculatory blood flow are crucial for novel therapeutics enhancing RBC function.

The transport of respiratory gases through the body depends on the oxygen‐carrying capacity of RBCs. Normal oxygen saturation of Hb is about 97%–100% [41]. Initial O_2_ binding alters Hb conformation, dissociating H^+^ and facilitating the cooperativity of the oxygen binding sites. Under acidic conditions, Hb's conformation changes, releasing oxygen while attaching protons as the molecule changes from a higher to a lower oxygen affinity state. The Hb oxy–deoxy conformational changes occur through allosteric interactions between O_2_ and H^+^/CO_2_ binding sites. In steady state, H^+^ is passively distributed across the RBC membrane, and intracellular pH changes are related to extracellular pH, Hb‐O_2_ saturation, and organic phosphate content [42]. Therefore, blood oxygen‐carrying capacity and regulation of oxygen delivery from Hb into tissues are controlled by the O_2_ tension gradient, temperature, blood pH, and 2,3‐diphosphoglycerate concentration [43, 44, 45, 46].

Physiological alterations in deoxygenated RBCs show decreased Hb‐oxygen saturation, binding of organic phosphates to deoxyHb [6, 47], an increase in pH due to the acid–base properties of deoxyHb [48, 49], decreased binding of Mg^2+^ by ATP, increased binding of ATP to deoxyHb [50] and alterations in glycolysis [39, 40]. The pH in deoxygenated cells increases by 0.14 pH units compared with oxygenated cells [28]. DeoxyHb is a stronger proton acceptor than oxyHb [48, 49], thus, intracellular pH indeed increases by 0.1–0.15 upon deoxygenation [51].

NMR reveals that RBC deoxygenation leads to down‐field shifts and line broadening of intracellular phosphate resonances in the ^31^P NMR spectra [29, 52, 53]. Chemical shifts change proportionally to Hb‐oxygen saturation. The 2,3‐DPG chemical shift changes in oxy‐ and deoxyHb solutions [54] and in oxygenated and deoxygenated red cells [51] are related to Hb‐2,3‐DPG. The 2,3‐DPG ^31^P‐signals shift down‐field because of the strong binding of 2,3‐DPG by deoxyHb [28, 55]. Meanwhile, the line broadening in deoxygenated RBCs results from a paramagnetic susceptibility gradient across the cell membrane, leading to magnetic field inhomogeneities. Although deoxyHb has a high‐spin paramagnetic Fe^2+^ ion, the paramagnetic shift effect is proportional to deoxyHb concentration [53]. Moreover, pH and 2,3‐DPG‐Hb interactions depend on deoxyHb concentration [28]. Therefore, both the down‐field shift of intracellular phosphate signals and line broadening indicate the binding of 2,3‐DPG to Hb in deoxygenated RBCs.

Here, we present results that clarify some aspects of RBC functions and interaction with ITPP. Its effects on RBCs were monitored by observing the 2,3‐DPG resonances in ^31^P NMR spectra to assess ITPP‐induced oxygenation of RBCs. Phosphate signals of the effector after treatment of RBCs with ITPP shifted down‐field and lines broadened. ITPP‐induced effects are similar to those reported for deoxygenated systems, indicating that ITPP‐treated RBCs are deoxygenated and the amount of deoxyHb has increased. NMR changes reflect the deoxygenation of RBCs upon treatment with ITPP. These results, as well as the function of ITPP as allosteric effector of Hb, justify the mechanism of ITPP action on RBCs.

Upon entering erythrocytes, ITPP affects their intracellular pH and oxygenation state. The compound possibly displays a higher affinity for Hb than the natural effector 2,3‐DPG. Therefore, ITPP may displace 2,3‐DPG from Hb. Assuming the similarity between these processes, ITPP binds to the central compartment of the Hb AA microenvironment, lowers oxyHb saturation, and increases the amount of bound effector. ITPP may improve the exchange properties of Hb. Under hypoxia, ITPP can reduce Hb affinity for oxygen, stabilise its deoxygenated state and, therefore, elevate oxygen release from erythrocytes. Thus, ITPP, modulating erythrocyte physiology, enhances the oxygen supply to tissues.

Ultimately, sustained increase in oxygen partial pressure (pO_2_) permitting vessel normalisation counteracts tissue hypoxia, confirming the medical potential of the ITPP application. ITPP‐mediated higher delivery of oxygen by RBCs, inducing vasculature remodelling into normal vessels, could restore a physiological level of pO_2_, reverse the pathologic angiogenesis and immunosuppression, facilitate the entry of immune cells into the tumour, and establish an efficient anti‐cancer immune response [3, 5]. Vessel normalisation driving tumour oxygenation reduces oedema and enhances the efficacy of cytotoxic anticancer drugs. This approach is known as a treatment of the hypoxia‐dependent diseases, such as cardiovascular defects [38].

The ^31^P resonances of 2,3‐DPG, with their distinct chemical shift values, constitute reporter groups of the intracellular pH and oxygenation state of the internal RBC microenvironment. Initially, we used the ^31^P measurements of the 3‐P and 2‐P chemical shifts of the 2,3‐DPG as indicators of the RBC internal pH. We have demonstrated that the pH inside the RBC can be deduced from a series of experiments using buffers with predefined pH values for the intact and lysed erythrocytes. The internal pH of RBCs was dependent on the pH of the external microenvironment in the absence of band‐3 inhibitors [56].

Our conclusions consider the fact that by lysing cells that contain various types of organelles it would be difficult to control the extent of the effects of the various organelles, especially endosomes, the pH of which is 4.5, if they were lysed, a difficult step to avoid during the lysis procedure. But erythrocytes are devoid of organelles (nucleus, ER, Golgi apparatus, mitochondria…) due to their elimination during maturation, allowing to reduce such a risk by the strict control and the comparative way the experiments are conducted to insure the data soundness. It is worth noting that ITPP impacts glycolysis in RBCs, as Band‐3 controls the activity of glycolytic enzymes its destabilisation permits the lowering of internal pH, thus stabilises deoxyHb [9]. Similarly, 2,3‐DPG lowers the oxygen affinity to Hb. Both the internal H^+^ concentration and 2,3‐DPG shift the conformational equilibrium of Hb towards the deoxy form, but with different effects on ^31^P resonances. Lowering internal pH causes the up‐field shift of the ^31^P resonances of 2,3‐DPG, while its interaction with Hb induces the down‐field shift. The overlap of these effects makes the measurements less straightforward.

As previously reported by Labotka, the pH changes are not the principal cause for the alterations of 2,3‐DPG chemical shifts but are mostly due to different magnetic susceptibility in RBCs as the amount of deoxyHb increases. Upon deoxygenation, deoxyHb binds strongly to 2,3‐DPG, thus, the down‐field shifts of 3‐P & 2‐P resonances are larger than these for P_i_. Moreover, the resonances broaden as the fraction of deoxyHb increases. Therefore, the down‐field shifts of 3‐P, 2‐P, and P_i_ resonances, and the observed line broadening primarily report the oxygenation states of the RBCs (Figure 2) [28].

Three pyrophosphates substitute inositol in ITPP; their ^31^P resonances can serve as distinct reporters (Figure 3) and should indicate the presence of ITPP in samples throughout in vitro and in vivo experiments. We expected that interactions with Hb would alter linewidth and resonance positions of the ITPP resonances in the microenvironment of the RBC. However, additional washing steps in analysis of the ITPP effects on RBCs pointed to the detection limits using the NMR experiments. Duarte et al. reported the amounts of ITPP used for stimulation and the ITPP uptake levels by RBCs [9], demonstrating that by RBC stimulation with 60, 120 and 240 mM ITPP the intracellular concentration of ITPP ranged 3.9–5.5 mmol/L. The estimated amounts for the up‐taken ITPP would be approximately 1.25–1.5 mg in 0.5 mL. By this estimate, approximately 2% of the ITPP used for the stimulation of the RBCs ended up inside. This important observation allows for the explanation of the second mechanism of ITPP action in circulation, involving the activation of the PTEN tumour suppressor and main regulator of angiogenesis that is selectively activated in endothelial cells [3, 17]. We confirmed here that ITPP is detected in circulation 1 h after intraperitoneal injection and is available for interaction with endothelial cells.

In our experiments, we used an ITPP concentration of 3 mg/mL for in vitro stimulation, thus, expecting ~30 μg in 0.5 mL of the RBC sample used for the NMR measurement. To define the NMR detection limits for ITPP in solution, ^1^H NMR and ^31^P experiments were acquired (Figure 5) and demonstrated ~150 μg in 0.5 mL as the detection limit for ITPP using ^1^H NMR. This limit was even higher for the ^31^P spectra, as the ^31^P sensitivity is approximately 7% of ^1^H. These observations explain the lack of NMR‐detectable ITPP in the in vitro and in vivo RBC samples after extensive washing.

## 4 Conclusions

The 2,3‐DPG ^31^P resonances (and P_i_) chemical shift values are pH‐dependent. Measurements on intact and lysed RBCs allow the determination of apparent internal pH and are effective indicators of the RBC oxygenation state.

ITPP affects the pH and oxygenation state of RBCs, manifested by the chemical shifts and NMR line‐broadening of 2,3‐DPG signals in the ^31^P NMR spectra.

ITPP acts via the band‐3 protein, and its effects are abolished by DIDS (band‐3 inhibitor). Therefore, 2,3‐DPG ^31^P resonances can probe the transmembrane ionic equilibrium.

NMR‐detectable ITPP resonances in RBCs in vitro and in vivo are concentration‐limited. ITPP increased the deoxy‐Hb in RBCs with a pH increase and a down‐field shift of 3‐P and 2‐P signals. DeoxyHb‐bound 2,3‐DPG line‐broadening of signals is deoxyHb concentration‐dependent.

Overall, the mechanism of action of ITPP may be dual: on the one hand, it may act by binding directly to the allosteric regulatory pocket of Hb, displacing a small amount of 2,3‐DPG; on the other hand, as indicated by the effect of DIDS, it interacts with the membrane band‐3 channel protein, thus, modulating the internal pH of RBCs and increasing oxygen release in a Bohr effect manner, which could be the dominant mode of action of ITPP.

## 5 Significance

The key challenge to permit the efficacy of treatment against all diseases that develop in low pO_2_ conditions, is to reach stable alleviation of hypoxia. Tumour growth induces hypoxia in the microenvironment and enhances angiogenesis to compensate for the local oxygen deprivation. Thus, a promising mode of cancer treatment is to use drugs that counteract hypoxia and normalise the aberrant vascularisation thanks to a more efficient release of oxygen by Hb in RBCs. Oxygen availability to cells in tissues depends on the O_2_ supplier ability to locally release oxygen. This is increased by *myo*‐inositol tris pyrophosphate, here ITPP action on RBCs is deciphered in vitro and in vivo. ITPP is shown to modify the intracellular pH and Hb oxygenation in RBC through its first interaction with band‐3 ion channel. ITPP mechanism is proven inside the RBC by its effects on 2,3‐DPG ^31^P resonances considering their sensitivity upon pO_2_ and pH. The ^31^P NMR signals of 2,3‐DPG in RBCs in situ serve as reporters of intracellular pH and oxygenation state of erythrocytes. ITPP acts as an effector of Hb that can change the glycolytic activity in the RBCs. It is capable of pH lowering and subsequently, enhances oxygen release. Moreover, NMR shows the rapid and stable availability of ITPP in the circulating blood and validates the ITPP‐based, vessel normalisation strategies against pathologic angiogenesis.

## 6 Methods

### 6.1 Mice

Female C57BL/6 mice were obtained from the Experimental Medicine Centre at the Medical University of Bialystok (Bialystok, Poland). Experiments were performed in accordance with European Union Directive 2010/63/EU for animal experiments and were approved by the 1st Local Ethics Committee for Experiments with the Use of Laboratory Animals, Wroclaw, Poland.

### 6.2 Preparation of the Intact RBC and Their Hemolysates

Whole blood was collected from the ophthalmic vein of healthy, female C57BL/6 mice subjected to anaesthesia with inhaled isoflurane (Abbott, Cat. No. B506). Freshly collected murine blood (1 mL) was split into four sample tubes (250 μL each) and suspended in the 30 mM HEPES buffer (containing 150 mM NaCl and 100 μM CaCl_2_) at desired pH values (6.8, 6.9, 7.0 and 7.2) and washed four times. Finally, the intact RBCs were suspended in 450 μL of 30 mM HEPES buffer at the desired pH in an Eppendorf tube. Fifty microlitres of D_2_O was added, followed by the transfer of the sample into a 5 mm NMR tube. After the measurement on the intact RBCs, the sample was transferred to an Eppendorf tube and sonicated at approximately 35 W for 20 s (twice) using a Bandelin Sonoplus model 2070 Sonifier. The lysed RBC sample was then transferred back into a new 5 mm NMR tube for the lysed RBC measurement. No additional D_2_O was added. All steps of the experiment were performed under standard laboratory conditions, ensuring a constant supply of atmospheric oxygen throughout the procedures. Between measurements, the samples were stored at 4°C. The actual pH for the intact RBC suspension and the lysed RBC were recorded. Biological repeats report data from groups of *n* = 6 mice.

### 6.3 Preparation of the DIDS‐Treated RBCs

DIDS is a reversible inhibitor of some membrane transporters, for example, anion transport in RBCs. It binds covalently to the RBC band‐3 anion exchanger. RBCs were treated with 4,4′‐Diisothiocyanatostilbene‐2,2′‐disulfonic acid (DIDS) by mixing blood with a 2 mM solution of DIDS (vol/vol) and incubating for 1 h at 37°C [9]. The RBCs were then washed by resuspension in saline and centrifugation (700 g, 5 min). All experiments were performed under standard laboratory conditions, ensuring a constant supply of atmospheric oxygen throughout the procedures. To prepare the samples for NMR analysis, we carefully transferred them to tightly sealed glass vials to prevent any gas exchange, subsequently storing them at 4°C until analysis.

### 6.4 Preparation of the RBC Hemolysates Following Treatment With ITPP

ITPP was kindly provided by Prof. J‐M. Lehn and had been prepared as described [3, 8]. For in vitro analyses of the effect of ITPP on RBCs, freshly collected murine blood (1 mL) was split into four sample tubes (250 μL each) and suspended in 30 mM HEPES buffer (containing 150 mM NaCl & 100 μM CaCl_2_, IIET PAS) at desired pH values (6.8, 6.9, 7.0, and 7.2) and washed four times. ITPP (5 μL of the stock solution, 300 mg/mL—final concentration of ITPP in the sample: 3 mg/mL) was added to 500 μL of each of the RBC suspensions in buffers with different pH‐values, followed by incubation at 37°C for 30 min. Fifty microlitres of D_2_O was added to the RBC preparations (450 μL) in the HEPES buffered solution described above, and the suspension was transferred to the NMR tubes. After the first measurement on intact RBCs, the sample was moved to an Eppendorf tube and washed three times with the corresponding buffer. The washed RBC samples were split into two Eppendorf tubes: one for the measurement of the washed‐RBC, and the other for the measurement of the lysed‐RBCs. The lysis was performed as described above. The samples were then transferred back to a 5 mm NMR tube for the re‐measurement of the washed‐RBC and the lysed‐RBC ^31^P spectra. To account for the ITPP content at different stages of the preparations, the supernatants were preserved for separate NMR measurements. All steps of the experiment were performed under standard laboratory conditions, ensuring a constant supply of atmospheric oxygen throughout the procedures. Between the measurements, the samples were stored at 4°C, and the actual pH was recorded for the intact RBC suspension and the lysed‐RBC.

### 6.5 Mice Treatment With ITPP and Whole Blood Collection

Eight‐ to ten‐week‐old female C57BL/6 mice were intraperitoneally (i.p.) inoculated with various doses of ITPP (1.5, 2.25, or 3.00 g/kg of body weight suspended in saline). The control group received NaCl. One hour later, blood samples were collected from each individual mouse into heparinised micro‐tubes. Blood samples were collected from the ophthalmic vein after anaesthesia by inhaled isoflurane (Abbott, Cat. No. B506), and the mice were sacrificed by cervical dislocation. Directly after collection, the pH was measured in each blood sample. All subsequent handling of the obtained RBC and the measurements were done as described for the in vitro experiments.

### 6.6 NMR Spectroscopy

NMR spectra of the intact RBC suspensions, the lysed‐RBC, and the supernatants were recorded for the buffered H_2_O solutions (max. volume ~500 μL) containing 10% D_2_O at 288 K on a Bruker Avance III NMR 600 MHz spectroscope equipped with the 5 mm ^1^H/^13^C/^15^N/^31^P Cryoprobe (QCI). 1D ^1^H and ^31^P spectra with proton decoupling were acquired. For the ^31^P spectra, typically 256 scans were accumulated and averaged. The spectral width used was 40 ppm. The spectra were phased and apodised by the exponential window function with the line broadening set to 10 Hz. The peak‐picking was done using an automatic built‐in Topspin ‘auto‐pick’ algorithm. The data were acquired and processed using Topspin software (Bruker Biospin). 85% H_3_PO_4_ was used as an external reference.

## Author Contributions

**Sabina Koj:** conceptualization (supporting), data curation (supporting), formal analysis (equal), investigation (equal), methodology (supporting), validation (equal), writing – original draft (equal), writing – review and editing (equal). **Tomasz Niedziela:** conceptualization (lead), data curation (equal), formal analysis (lead), investigation (equal), methodology (equal), software (equal), supervision (lead), validation (equal), visualization (lead), writing – original draft (lead), writing – review and editing (equal). **Joanna Rossowska:** conceptualization (supporting), data curation (supporting), formal analysis (supporting), investigation (equal), methodology (lead), resources (supporting), supervision (supporting), validation (supporting), writing – original draft (supporting), writing – review and editing (supporting). **Jean‐Louis Schmitt:** validation (supporting). **Jean‐Marie Lehn:** conceptualization (equal), resources (equal), validation (equal), writing – review and editing (equal). **Claude Nicolau:** conceptualization (equal), validation (equal), writing – review and editing (equal). **Claudine Kieda:** conceptualization (lead), data curation (lead), formal analysis (equal), funding acquisition (lead), investigation (lead), methodology (equal), project administration (lead), resources (lead), software (equal), supervision (equal), validation (equal), visualization (supporting), writing – original draft (equal), writing – review and editing (equal).

## Conflicts of Interest

The authors declare no conflicts of interest.

## Supporting information


Tables S1–S3.

## Data Availability

All data associated with this study are available within the article and its Supporting Information file. Raw data are available upon request from the corresponding authors.
