# Supplementary material for: Modulation of the Oxygenation State and Intracellular pH of Erythrocytes by Inositol‐Trispyrophosphate Investigated by 31P NMR Study of 2,3‐DPG
Source: J Cell Mol Med. 2025 Jan 19;29(2):e70343. doi: 10.1111/jcmm.70343 (PMC11742965; doi:10.1111/jcmm.70343)
Supplement: Supplementary file 1 [file JCMM-29-e70343-s001.odt]

Supplementary Information for

MODULATION OF OXYGENATION STATE AND INTRACELLULAR pH OF ERYTHROCYTES BY INOSITOL TRISPYROPHOSPHATE INVESTIGATED BY 31P NMR STUDY OF 2,3-DPG

Sabina Koj1*, Tomasz Niedziela1*#, Joanna Rossowska1, Jean-Louis Schmitt2, Jean-Marie Lehn2, Claude Nicolau3,4, Claudine Kieda5,6#

Author affiliations:

1Hirszfeld Institute of Immunology and Experimental Therapy, Polish Academy of Sciences, 53-114 Wroclaw, Poland

2ISIS – University of Strasbourg, F-67000 Strasbourg

3NormOxys Inc., Boston, MA 02135, USA

4Friedman School of Nutrition Science and Policy, Tufts University, Boston, MA 02111, USA

5Centre for Molecular Biophysics, UPR CNRS 4301, 45071 Orleans Cedex 2, France

6Laboratory of Molecular Oncology and Innovative Therapies, Military Institute of Medicine – National Research Institute, 04-141 Warsaw, Poland

*These authors equally contributed to the work

#Correspondence should be addressed to email: 

[claudine.kieda@cnrs-orleans.fr](mailto:claudine.kieda@cnrs-orleans.fr), 

[tomasz.niedziela@hirszfeld.pl](mailto:tomasz.niedziela@hirszfeld.pl)

Table 1. Chemical shift values of 3-P(DPG), 2-P(DPG) and Pi resonances in the 1D NMR 31P spectra of intact and lysed RBC at pH values of 6.8, 6.9, 7.0 and 7.2.

|  | RBC | 31P Chemical shifts [ppm] |
| --- | --- | --- |
|  | pH |  | 3-P(DPG) | 2-P(DPG) | Pi |
| 1. | 6.8 | intact | 3.202 | 2.277 | 1.794 |
|  |  | lysed | 3.088 | 2.089 | 1.747 |
| 2. | 6.9 | intact | 3.26 | 2.36 | 1.903 |
|  |  | lysed | 3.172 | 2.200 | 1.868 |
| 3. | 7.0 | intact | 3.321 | 2.452 | 1.996 |
|  |  | lysed | 3.241 | 2.289 | 1.983 |
| 4. | 7.2 | intact | 3.436 | 2.628 | 2.167 |
|  |  | lysed | 3.376 | 2.494 | 2.185 |

Table 2. Chemical shift values of the 3-P(DPG), 2-P(DPG) and Pi resonances in the 1D NMR 31P spectra of intact RBC, ITPP-treated RBC, ITPP-treated RBC washed, intact RBC in the presence of the band-3 inhibitor (DIDS), and the ITPP-treated RBC in the presence of DIDS; at  pH values of 6.8, 6.9, 7.0 and 7.2.

|  | RBC | 31P Chemical shifts [ppm] |
| --- | --- | --- |
|  | pH |  | 3-P(DPG) | 2-P(DPG) | Pi |
| 1. | 6.8 | Intact (Control) | 3.19 | 2.27 | 1.77 |
|  |  | +ITPP | 3.22 | 2.32 | 1.79 │ 1.74* |
|  |  | +ITPP washed | 3.25 | 2.35 | 1.78 │ 1.74* |
|  |  | +DIDS | 3.44 | 2.63 | 2.19 |
|  |  | +ITPP+DIDS | 3.45 | 2.64 | 2.20 |
| 2. | 6.9 | intact | 3.24 | 2.35 | 1.87 |
|  |  | +ITPP | 3.28 | 2.41 | 1.87 |
|  |  | +ITPP washed | 3.31 | 2.41 | 1.87 |
|  |  | +DIDS | 3.44 | 2.63 | 2.23 |
|  |  | +ITPP+DIDS | 3.45 | 2.67 | 2.21^ |
| 3. | 7.0 | intact | 3.32 | 2.43 | 1.97 |
|  |  | +ITPP | 3.34 | 2.47 | 1.98 |
|  |  | +ITPP washed | 3.35 | 2.48 | 1.99 |
|  |  | +DIDS | 3.46 | 2.68 | 2.23 |
|  |  | +ITPP+DIDS | 3.45 | 2.69 | 2.16 |
| 4. | 7.2 | intact | 3.41 | 2.60 | 2.14 |
|  |  | +ITPP | 3.45 | 2.65 | 2.19 │ 2.14* |
|  |  | +ITPP washed | 3.44 | 2.62 | 2.19 │ 2.14* |
|  |  | +DIDS | 3.49 | 2.74 | 2.26 |
|  |  | +ITPP+DIDS | 3.49 | 2.73 | 2.27 |

* The signal appeared as two distinct resonances of Pi; ^ Assigned manually (a low intensity, broad resonance)

Table 3. Chemical shift values of the 3-P(DPG), 2-P(DPG) and Pi resonances in the 1D NMR 31P spectra of RBC isolated from mice after treatment ip with ITPP at amounts 30 mg, 45 mg, and 60 mg.

|  | ITPP [mg] | RBC | 31P Chemical shifts [ppm] |
| --- | --- | --- | --- |
|  |  |  | 3-P(DPG) | 2-P(DPG) | Pi |
| 1. | 30 | intact | 3.634 | 2.987 | 2.517 |
|  |  | washed | 3.327 | 2.456 |  |
|  |  | lysed | 3.555, 3.247 | 2.309 | 1.94 |
| 2. | 45 | intact | 3.682 | 3.012 | 2.55 |
|  |  | washed | 3.328 | 2.458 |  |
|  |  | lysed | 3.247 | 2.305 | 1.966 |
| 3. | 60 | intact | 3.639 | 2.957 | 2.51 |
|  |  | washed | 3.334 | 2.469 | 2.03 |
|  |  | lysed | 3.232 | 2.274 | 1.995 |
